# Predictions of Lattice Parameters in NiTi High-Entropy Shape-Memory Alloys Using Different Machine Learning Models

**DOI:** 10.3390/ma17194754

**Published:** 2024-09-27

**Authors:** Tu-Ngoc Lam, Jiajun Jiang, Min-Cheng Hsu, Shr-Ruei Tsai, Mao-Yuan Luo, Shuo-Ting Hsu, Wen-Jay Lee, Chung-Hao Chen, E-Wen Huang

**Affiliations:** 1 Department of Materials Science and Engineering, National Yang Ming Chiao Tung University, 1001 University Road, Hsinchu 30010, Taiwan; lamtungoc1310@nycu.edu.tw (T.-N.L.); tidew0915@gmail.com (M.-C.H.); owo8691@gmail.com (M.-Y.L.); sthsu.en12@nycu.edu.tw (S.-T.H.); 2 Department of Physics, College of Education, Can Tho University, Can Tho City 900000, Vietnam; 3 Electrical and Computer Engineering Department, Old Dominion University, Norfolk, VA 23529, USA; jjian001@odu.edu; 4 Computer Science and Information Engineering, National Yang Ming Chiao Tung University, 1001 University Road, Hsinchu 30010, Taiwan; srt093.cs10@nycu.edu.tw; 5 National Center for High-Performance Computing, Taichung City 40763, Taiwan; wjlee@narlabs.org.tw; 6 High Entropy Materials Center, National Tsing Hua University, Hsinchu 30013, Taiwan; 7 Department of Materials Science and Engineering, Case Western Reserve University, Cleveland, OH 44106, USA

**Keywords:** machine learning, linear regression, random forest, support vector regression, shape-memory alloys

## Abstract

This work applied three machine learning (ML) models—linear regression (LR), random forest (RF), and support vector regression (SVR)—to predict the lattice parameters of the monoclinic B19′ phase in two distinct training datasets: previously published ZrO_2_-based shape-memory ceramics (SMCs) and NiTi-based high-entropy shape-memory alloys (HESMAs). Our findings showed that LR provided the most accurate predictions for a_c_, a_m_, b_m_, and c_m_ in NiTi-based HESMAs, while RF excelled in computing β_m_ for both datasets. SVR disclosed the largest deviation between the predicted and actual values of lattice parameters for both training datasets. A combination approach of RF and LR models enhanced the accuracy of predicting lattice parameters of martensitic phases in various shape-memory materials for stable high-temperature applications.

## 1. Introduction

The most widely used NiTi-based shape-memory alloys (SMAs) are known for their good shape memory and superelastic characteristics, based on the transformation between austenite and martensite phases [1,2]. However, their low martensitic transformation temperatures (TTs) below 100 °C are the most common challenges for high-temperature applications [3]. A composition engineering strategy in adding more alloying elements of Pt, Pd, Co, Cu, Zr, and Hf is believed to significantly increase TTs in NiTi-based high-entropy SMAs (HESMAs) [4,5,6]. In addition to high TTs, small thermal hysteresis is expected for stable high-temperature applications of the NiTi-based HESMAs.

The application of machine learning (ML) techniques has garnered significant attention for their potential in predicting and designing alloy compositions with optimized structural and functional properties [7,8,9,10,11]. Xue et al. applied different ML models to predict the transformation temperatures in NiTi-based SMAs [12]. Several ML approaches such as linear regression (LR) [9,13], random forest (RF) [11,14], neural network (NN) [11,15], and support vector regression (SVR) [11,16], have been commonly employed for computational prediction of crystal structures. Li et al. employed the MLatticeABC algorithm, a random forest model, to predict the lattice constants of crystal materials [11]. Pang et al. have recently proposed a linear regression (LR) model to successfully compute the lattice parameters of monoclinic and tetragonal structures in ZrO_2_-based shape-memory ceramics (SMCs) [9]. Their approach has shed light on the composition design of new martensitic materials with desirable low thermal hysteresis and high TTs. Consequently, we are highly motivated to extend their promising strategy to our research field on the NiTi-based HESMAs.

The present study aimed to identify the most effective ML model for accurately predicting the lattice parameters of a monoclinic structure. We first followed previously published research to forecast the lattice parameters of monoclinic structures from a public training dataset of ZrO_2_-based SMCs [9] using two more nonlinear models of random forest (RF) and support vector regression (SVR), besides LR. We then applied these three various ML models to the training dataset of the NiTi-based HESMAs to evaluate the most effective ML approach in predicting all lattice parameters of monoclinic B19′ (a_m_, b_m_, c_m,_ and β_m_) and cubic B2 (a_c_) structures.

## 2. Materials and Methods

Three distinct ML models were applied to predict the lattice parameters in the two training datasets of ZrO_2_-based SMCs and NiTi-based HESMAs. LR, a fundamentally predictive method in statistics, is suitable for datasets with a roughly linear relationship between variables, and it can be either simple or multivariate. RF and SVR are considered for datasets with nonlinear relationships. RF is an ensemble learning method composed of numerous decision trees, with the final output being an aggregation of the individual trees’ predictions. This method is highly effective in evaluating feature importance, handling high-dimensional data, managing missing values, and demonstrating robust performance in diverse and unbalanced datasets. Therefore, RF enhances model diversity through random feature selection, effectively minimizing overfitting and increasing predictive accuracy. Conversely, SVR functions by mapping data to a high-dimensional space to identify the optimal fitting line or hyperplane. Employing kernel functions, SVR allows the construction of nonlinear relationships within the original feature space. A distinctive aspect of SVR is its ε-insensitivity band, tolerating minor prediction deviations without penalty, making it ideal for datasets with intricate or nonlinear associations. SVR’s regularization ensures strong generalization capabilities, effectively preventing overfitting. Both linear and nonlinear issues can be adeptly managed by selecting appropriate kernel functions. Like RF, SVR remains potent in scenarios where feature count surpasses sample size, ensuring reliable model predictions.

The feature for each data point was modeled as follows [9]:

(1) $$x^{ave}=\sum_{i}f^{i}x^{i}$$

where *x^ave^* is the weighted average of all constituent elements, *f^i^* is the mole fraction of a specific element *i*, and *x^i^* is the feature value of element *i*.

**Figure 1:**
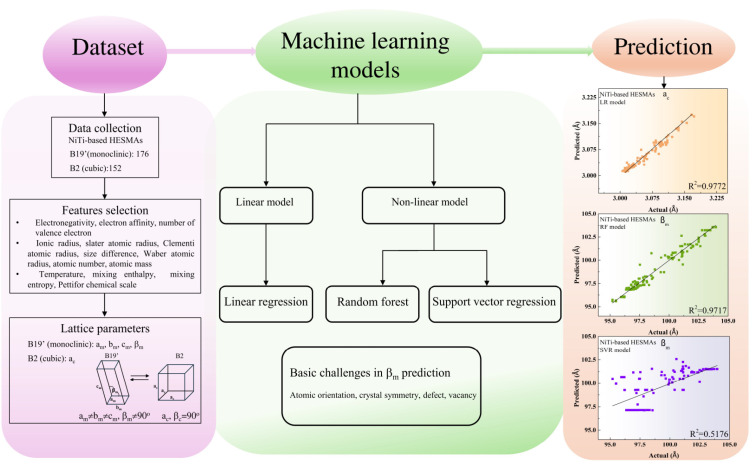
Flowchart for the computational prediction of lattice constants in the NiTi-based HESMAs.

Our training dataset of NiTi-based HESMAs included data points of 152 cubic and 176 monoclinic structures comprising constituent compositions of Ni, Ti, Hr, Hf, Cu, Fe, and Co in the Ni_50-x-y-z_Ti_50-h-k_Hf_h_Zr_k_Cu_x_Fe_y_Co_z_, which was collected from previously published studies [4,17,18,19,20,21,22,23,24,25,26,27,28,29,30,31,32,33,34,35,36,37,38,39,40,41,42,43,44]. A set of 14 input features were electronegativity, electron affinity, number of valence electrons, Pettifor chemical scale, ionic radius, slater atomic radius, Clementi atomic radius, size difference, Waber atomic radius, atomic number, atomic mass, temperature, mixing enthalpy, and mixing entropy. The test root mean square error (RMSE) during cross-validation of the best feature set was used to evaluate the accuracy of ML models. Figure 1 illustrates the procedure for the computational prediction of lattice constants in the NiTi-based HESMAs.

## 3. Results

**Figure 2:**
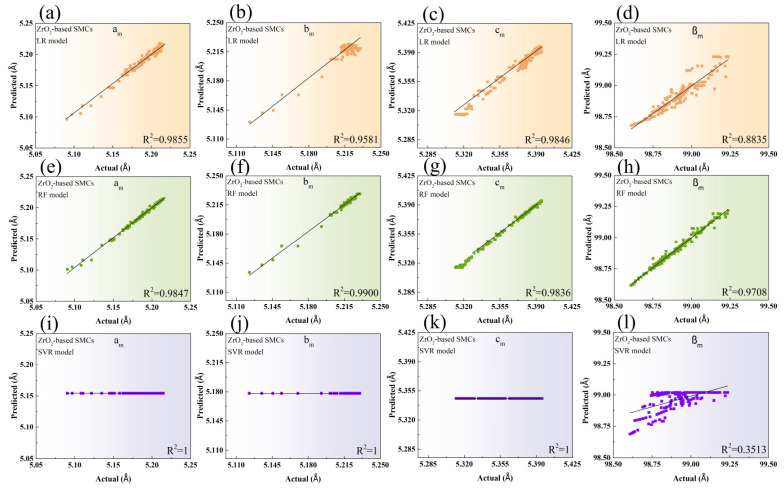
The predicted versus actual lattice constants of a_m_ (**a**), b_m_ (**b**), c_m_ (**c**), and β_m_ (**d**) modeled by LR. Those of a_m_ (**e**), b_m_ (**f**), c_m_ (**g**), and β_m_ (**h**) modeled by RF. Those of a_m_ (**i**), b_m_ (**j**), c_m_ (**k**), and β_m_ (**l**) modeled by SVR in the ZrO_2_-based SMCs. The solid black line depicts the perfect match between the predicted versus actual values of lattice constants.

In accompanying LR used in published research [9], we applied two more different nonlinear models of RF and SVR to the public training dataset of ZrO_2_-based SMCs to explore the most appropriate model in predicting the lattice parameters of monoclinic structure. Figure 2 shows the predicted versus actual lattice parameters fitted using three distinct models. A comparison of the test RMSE among the three ML models is presented in Table 1. Lower test RMSE values indicate higher accuracy between the predicted and actual values. The test RMSE values of a_m_, b_m_, c_m,_ and β_m_ modeled by LR in the present work were in good accordance with those obtained in previously published work [9], seen in Table 1, demonstrating the reliability of our predictions in modeling the lattice parameters of the monoclinic phase.

The LR in Figure 2a–d revealed a closer fit between the predicted and actual values for a_m_ and c_m_, in agreement with R^2^ values. The RF in Figure 2e–h showed a better match for b_m_ and β_m_, as also seen in the test RMSE and R^2^. It is noted that a lower accuracy of b_m_ and β_m_ than a_m_ and c_m_ by LR was found to be modeled more accurately by RF. The predicted values of a_m_, b_m_, and c_m_ could not be distinguished from their actual corresponding values, and a very high value of the test RMSE in predicting β_m_ was obtained by SVR, suggesting that SVR was not suited to compute the lattice parameters of monoclinic structures, shown in Figure 2i–l and Table 1.

Both LR and RF were found to be more suitable ML models for forecasting all lattice parameters of ZrO_2_-based SMCs, as there was a negligible discrepancy in the test RMSE between them. However, RF demonstrated higher accuracy in predicting b_m_ and β_m_ compared to LR, as evidenced by its lower test RMSE and higher R^²^ values.

**Figure 3:**
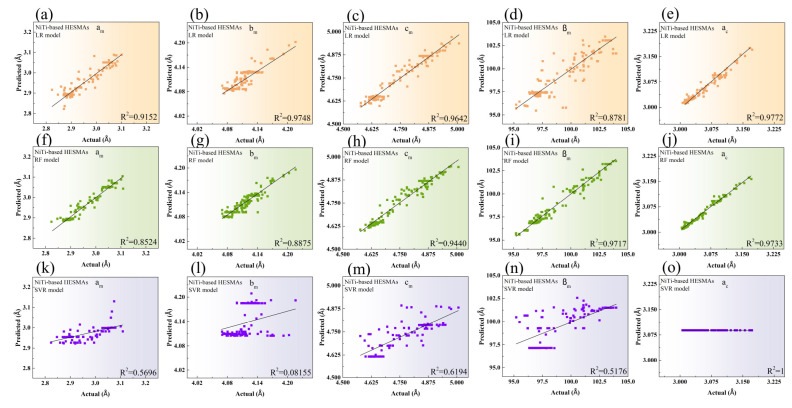
The predicted versus actual lattice constants of a_m_ (**a**), b_m_ (**b**), c_m_ (**c**), β_m_ (**d**), and a_c_ (**e**) modeled by LR. Those of a_m_ (**f**), b_m_ (**g**), c_m_ (**h**), β_m_ (**i**), and a_c_ (**j**) modeled by RF. Those of a_m_ (**k**), b_m_ (**l**), c_m_ (**m**), β_m_ (**n**), and a_c_ (**o**) modeled by SVR in the NiTi-based HESMAs. The solid black line depicts the perfect match between the predicted versus actual values of lattice constants.

To verify the potential of LR and RF ML models, we employed them to forecast the lattice parameters of monoclinic structures in the NiTi-based HESMAs in Figure 3. The predicted values of a_m_, b_m_, c_m_, and a_c_ exhibited the best match with the actual values when using LR, as illustrated in Figure 3a–e. β_m_ demonstrated the highest accuracy between the predicted and actual values using RF, as shown in Figure 3f–j and corroborated by the test RMSE values in Table 1. The remarkable accuracy of RF in predicting β_m_ in the NiTi-based HESMAs was also observed in the ZrO_2_-based SMCs. Conversely, SVR exhibited the poorest accuracy in modeling all lattice parameters of the monoclinic phase, as shown in Figure 3k–n. Moreover, no distinguishable prediction of a_c_ was computed by SVR in Figure 3o, which was similar to the predicted values of a_m_, b_m_, and c_m_ by SVR in the ZrO_2_-based SMCs (Figure 2i–k). A trivial discrepancy of experimental lattice parameters between data points was ascribed to the predicted values indistinguishable from the actual values in SVR.

**Figure 4:**
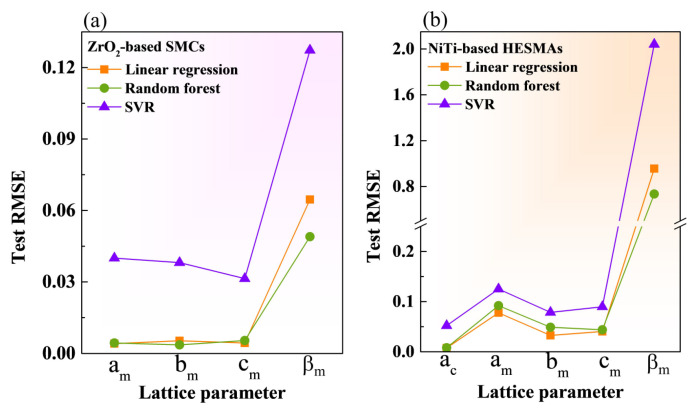
The test RMSE values among three ML models in computing the predicted lattice parameters of a_m_, b_m_, c_m_, and β_m_ in the ZrO_2_-based SMCs (**a**). Those of a_c_, a_m_, b_m_, c_m_, and β_m_ in the NiTi-based HESMAs (**b**).

As shown in Figure 4, the lowest test RMSE values for β_m_ were achieved in both datasets, indicating that RF is the most effective approach for predicting β_m_. Superior prediction of lattice angles using RF model has also been reported [11]. The test RMSE values of a_m_, b_m_, c_m_, and a_c_ in LR were slightly lower than those in RF, especially in the training dataset of the NiTi-based HESMAs, suggesting the superiority of the LR model in computing a_m_, b_m_, c_m_, and a_c_.

## 4. Conclusions

Among the three various ML approaches, the LR and RF models achieved lower test RMSE values and better prediction performance of the lattice constants for both monoclinic and cubic phases in the NiTi-based HESMAs. There was a better match between the predicted and actual values of a_m_, b_m_, c_m_, and a_c_ using LR in the NiTi-based HESMAs, which was also demonstrated in the published dataset of ZrO_2_-based SMCs. Meanwhile, greater accuracy of the β_m_ was found using the RF model in both the ZrO_2_-based SMCs and NiTi-based HESMAs. The concurrent combination of LR and RF models is expected to attain the highest accuracy in predicting all the unit-cell edge lengths and the inclination angles of the crystal structures. These initial predictive results are very crucial to the compositional design of new NiTi-based HESMAs with large recoverable strain and low density for their stable and cost-effective high-temperature applications in biomedicine, actuators, and the aerospace industry.

## Figures and Tables

**Table 1:** A comparison of test RMSE values among three ML models in predicting the lattice parameters of the ZrO_2_-based SMCs and NiTi-based HESMAs.

Materials	Phase	Predicted Lattice Parameters	ML Model	Test RMSE	Test RMSE [9]
ZrO_2_-based SMCs	Monoclinic (B19′)	a_m_	Linear regression	0.0041	0.0041
			Random forest	0.0044	
			Support vector regression	0.04	
	Monoclinic (B19′)	b_m_	Linear regression	0.0053	0.0053
			Random forest	0.0036	
			Support vector regression	0.0381	
	Monoclinic (B19′)	c_m_	Linear regression	0.0044	0.0045
			Random forest	0.0054	
			Support vector regression	0.0314	
	Monoclinic (B19′)	β_m_	Linear regression	0.0646	0.066
			Random forest	0.049	
			Support vector regression	0.1273	
NiTi-based HESMAs	Monoclinic (B19′)	a_m_	Linear regression	0.0777	
			Random forest	0.0919	
			Support vector regression	0.1252	
	Monoclinic (B19′)	b_m_	Linear regression	0.0326	
			Random forest	0.0488	
			Support vector regression	0.0785	
	Monoclinic (B19′)	c_m_	Linear regression	0.0403	
			Random forest	0.0437	
			Support vector regression	0.0896	
	Monoclinic (B19′)	β_m_	Linear regression	0.9571	
			Random forest	0.7355	
			Support vector regression	2.0401	
	Cubic (B2)	a_c_	Linear regression	0.0076	
			Random forest	0.0081	
			Support vector regression	0.0520	

## Data Availability

The original contributions presented in the study are included in the article, further inquiries can be directed to the corresponding authors.
